# Validation and psychometric testing of the Chinese version of the prenatal body image questionnaire

**DOI:** 10.1186/s12884-024-06281-w

**Published:** 2024-02-01

**Authors:** Qiaosong Wang, Jingjing Lin, Qirong Zheng, Liping Kang, Xueling Zhang, Kun Zhang, Rong Lin, Rongjin Lin

**Affiliations:** 1 School of Nursing, Fujian Medical University, Fuzhou City, China; 2 The First Affiliated Hospital of Fujian Medical University, Fuzhou City, China; 3 Quanzhou First Hospital Affiliated to Fujian Medical University, Quanzhou City, China

**Keywords:** Body image, Pregnancy, Assessment, Translation, Psychometrics, Validation

## Abstract

**Background:**

The body image during pregnancy potentially affects both short- and long-term maternal and child health outcomes, including pregnancy mood, postpartum weight recovery, and the quality of mother–child interactions. However, research on the impact of body changes during pregnancy in the Chinese population is scarce. A comprehensive, practical, and reliable tool for assessing pregnant women is needed to detect, intervene in, and implement the reduction of physical dissatisfaction risk. This study translated the Prenatal Body Image Questionnaire (PBIQ) into the Chinese version (PBIQ-C) to assess the body image of pregnant women and evaluated its reliability and validity.

**Methods:**

An improved Brislin translation model was used for the translation. A panel of experts determined the content validity. A convenience sample of 429 pregnant women was chosen from three third-class hospitals in different regions of Fujian Province, China. Factor analysis, Pearson’s correlation, retest reliability, and Cronbach’s alpha were employed to evaluate structural validity and reliability.

**Results:**

The final PBIQ-C had five dimensions with 21 items. Exploratory factor analysis obtained a five-factor solution, which accounted for a total of 60.34%. Confirmatory factor analysis showed that the model fit of the five-factor model also reached a satisfactory model fit after modifying: The Comparative Fit Index was 0.93, and the Tucker-Lewis Index was 0.92; the Root Mean Square Error of Approximation was 0.079. The content validity index of the scale ranged from 0.63 ~ 1.00. The Cronbach’s alpha coefficient was 0.95 for the total scale, and the test–retest reliability was 0.80.

**Conclusions:**

The findings indicated that the PBIQ-C is a valid and reliable instrument for assessing women’s body image during pregnancy, which helps in the early identification of body dissatisfaction during pregnancy and enables the early prevention of postpartum depression.

**Supplementary Information:**

The online version contains supplementary material available at 10.1186/s12884-024-06281-w.

## Background

Body image (BI) is an integral part of the self-concept, which refers to an individual's cognition, feelings, and behavioral intentions toward their body [1]. BI was coined by Head, a sixteenth-century psychiatrist who first used the term to describe the state of human bodily perception. In subsequent studies, the study of BI was extended to include the cognitive aspects of body representations formed by individuals in interpersonal interactions [2]. As research on BI in aesthetics, psychology, and medicine has advanced, its concept has expanded to include multiple dimensions resulting in a more comprehensive and refined understanding. The definition of BI proposed by Cash and other scholars is widely used in various fields of research today and is multidimensional [3]. The experience and perception of BI influence the quality of life, requiring us to adjust to change.

Owing to the influence of public opinion, people now experience increasing demands on their BI, which leads to certain cognitive biases and negative emotions, such as anxiety, depression, feelings of inferiority, and low self-esteem; and to changes in their BI through poor dietary habits, drug abuse, and excessive plastic surgery [4–6]. Such misperceptions and behaviors not only affect people’s psychological health but also cause damage to their physical and physiological functions. Body image disturbance (BID) is prevalent across all ages and life stages in both males and females [7]. From an evolutionary psychological perspective, females appear to be more prone to BID than males [3]. Approximately 85% of women experience varying degrees of BID [8], and other studies also show that women at all ages feel dissatisfied with their bodies [9].

In the past few decades, research on BI has developed rapidly, and researchers have devised numerous measures to assess BI. This has caused confusion in clinical decision-making regarding which BI-related scales to use [9]. Many BI measurements are applied in clinical practice, such as those for eating disorders, cancer, and the appearance of physical deformities (such as burns, cleft lip, palate, etc.) [10]. BI research has been developed in various fields; however, pregnant women are less studied. Pregnancy is a period in a woman's life during which a series of inevitable physiological changes, such as changes in body shape, weight gain, and stretch marks occur. The continued development of BID results in anxiety and depression, even affects the quality of mother–child interactions, posing a risk to maternal and child health [11]. BID in pregnant women is also associated with eating disorders and weight control, which are risk factors for adverse maternal and infant outcomes [12, 13]. Moreover, pregnant women may feel embarrassed or dissatisfied with their appearance owing to BID, and thus be reluctant to breastfeed [14]. Conversely, a positive side to maternal perceptions of body shape exists. For example, unlike the stigma associated with weight gain during non-pregnancy, weight gain during pregnancy is normal and desirable [15, 16]. Therefore, women may reevaluate their BI during pregnancy and constantly assess the value associated with their appearance to adapt to their changing bodies [16, 17]. Body self-assessment can lead to a series of changes in various aspects of pregnancy, including the perception of the ideal body shape, behavioral changes, emotional changes in appearance management, and a potential focal shift from physical appearance to body function. Most pregnant women are able to correctly reassess their bodies correctly and adjust their psychological state; however, some report an inability to adapt to such changes [15].

Current tools for assessing BI in pregnant women are limited to evaluating their satisfaction with their appearance and ignoring the sociocultural contextual factors of this particular population. Meireles et al. [18] summarized 40 studies and reported inconclusive data on body dissatisfaction in pregnant women, with contradictory results possibly related to different tools for measuring BI. The impact of BID on maternal and infant health requires, further investigations, particularly to develop specific instruments for assessing maternal BI. Thus, the applicability of tools for evaluating BI in pregnant women has been questioned, and a tool that can comprehensively and effectively assess BI in pregnant women is needed. The problem with BID is widespread, and there is an urgent requirement for research tools with specificity, multiple perspectives, and assessment dimensions and psychometric measures to assess pregnant women. Such tools will allow us to more accurately and promptly prepare for further interventions.

The Prenatal Body Image Questionnaire (PBIQ), developed by Professor Sohrabi in 2019, allows for a culturally specific assessment of a woman’s BI during pregnancy, incorporating local Iranian characteristics [19]. The PBIQ includes seven dimensions rated on a 5-point Likert scale, with higher total scores suggesting higher levels of dysfunctional BI. Regarding ethnic-cultural diversity, different ethnic groups have different customs and religious beliefs. Some ethnic groups subscribe to concepts such as body shame, clothes with full-body coverage, and sexual shame during pregnancy. In Confucian or Buddhist cultures, the body is considered part of human nature and reflects a person’s soul; bodily appearance is key in social comparisons and indicates belonging to a given level in the hierarchical society. Thus, an unkempt appearance invokes body shame [20]. Sexual shame during pregnancy can affect sexual function which may be associated with the religious and socio-cultural structure of the country in which an individual lives [21, 22]. It was found that 40% of Iranian women never talk about sexual issues with their partner, which can be attributed to the belief that women cannot discuss a topic of sexual issues unless asked by their husbands because of religious and social pressure [21]. Although Chinese women are also conservative in their sexual behavior, there are some cultural differences between China and Iran [23]. Therefore, the BI scale requires higher cultural adaptability and compatibility. In this study, we translate the PBIQ into Chinese (PBIQ-C) and adapt it to the Chinese cultural context based on the comments of obstetrics experts.

## Methods

### Study design

This study was conducted in two phases. In the first phase, the PBIQ was translated into Chinese and adapted to the Chinese cultural context. In the second phase, the psychometric properties of the PBIQ-C were assessed and validated.

### Participants and setting

A questionnaire was conducted from April 2022 to November 2023. The participants were a convenience sample of pregnant women from three third-class hospitals in Fujian Province, China, who were undergoing regular antenatal examinations. A paper or electronic version of the questionnaire was used to assess the pregnant women at the time of their visit or admission. The questionnaire included general demographic information and the PBIQ-C. Inclusion criteria were as follows: women in the gestation period; pregnant women aged ≥ 18, with good verbal and written communication skills; women who provided informed consent to participate in the study. The exclusion criteria were as follows: pregnant women who could not read or complete the questionnaire by themselves, and pregnant women with concurrent psychiatric or neurological disorders.

The sample size was determined based on a subject-to-item ratio of 5 to 10:1 and the 29-items questionnaire [24]. The sample size ranged from 290 to 580. In total, 429 pregnant women were recruited for this study. After this initial sample, 25 participants were asked to complete the PBIQ-C again after two weeks to evaluate retest reliability.

### Instruments

The PBIQ is a 30-item scale developed by Sohrabi et al. to comprehensively assess the BI of pregnant women [19]. The PBIQ comprises seven dimensions: (1) Fitness and beauty (4 items); (2) Lower body fat (5 items); (3) Attention to changes in pregnancy (5 items); (4) Shame (4 items); (5) Sexual attractiveness (4 items); (6) Negative feelings about skin changes (4 items), and (7) Symbol of motherhood (4 items). Both positive and negative wordings were used (items 20, 27, 28, 29, and 30 were reversely scored, whereas the rest were forward scored). Each item was rated on a five-point Likert scale (1 = strongly disagree to 5 = strongly agree). The total and dimension scores were the sum of the scores of each item, with the total score ranging from 30 to 150. A higher score on this questionnaire reflects greater dissatisfaction with BI during pregnancy. The original scale had acceptable internal consistency, with a Cronbach’s alpha of 0.925 for the overall scale and 0.721–0.887 for the subscales.

### Translation

**Fig. 1:**
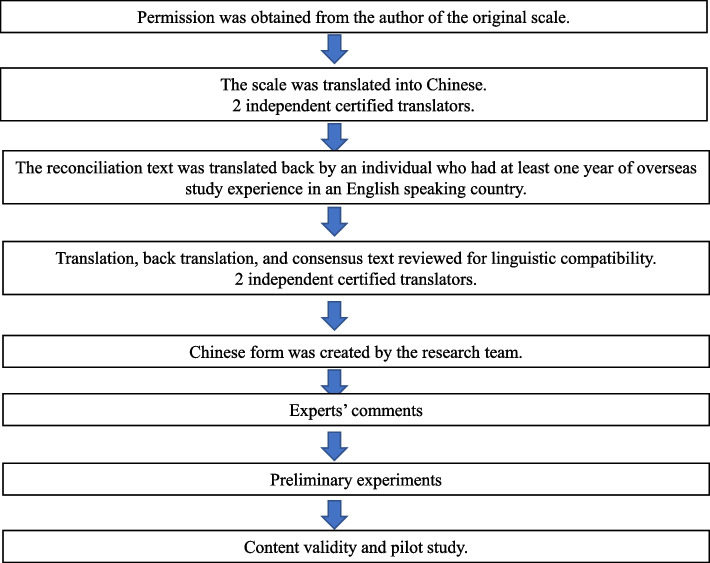
Translation and validity stages

We obtained permission from the original authors for translation and use before the study began. The translation process followed a modified Brislin translation model, which contained forward translation, back translation, and revision [25]. The original version of the PBIQ was independently translated by two graduate nursing students who were proficient in English and had passed the CET-6. The Chinese version was independently translated back into English by two researchers with over one year of study experience in English-speaking countries, both of whom were unfamiliar with the original PBIQ but proficient in English. Finally, two bilingual experts were invited to compare the translated versions with the original scales. Items with significant differences were retranslated. While checking the wording used, these experts also examined the cross-cultural adaptation needs of the sentences, as differences may lead to misunderstanding the question being asked [26]. We collected information about possible problems and suggestions, the ambiguous and complex items from the participants’ understanding of the contents of the scale and experts’ comments. The researchers continued to make modifications and adjustments to produce the PBIQ-C. Figure 1 summarizes this process.

### Experts’ comments

Eight obstetrics experts were invited, including six doctors and two midwives. Each expert evaluated the translation accuracy, content comprehension and cultural consistency of each item based on their theoretical knowledge and practical experience. The researcher held detailed discussions with these experts and summarized their comments.

### Prediction test

To determine the respondents’ understanding of the questionnaire, a preliminary survey was conducted using a convenience sample of 30 pregnant women admitted to the obstetric ward of a third-class hospital. After the survey and communication with the respondents, the items in the questionnaire that were difficult to understand or answer were marked, and the questions and suggestions made by the respondents were recorded. Based on the experts' suggestions and respondents' feedback, the PBIQ was revised to form the final Chinese version.

### Measures

The research team obtained research approval from the relevant authorities and conducted uniform training for the five research team members. The research tools included general information on the pregnant women, including age, BMI, educational level, place of residence, household income, career information, type of pregnancy and gestational age. Before commencing the survey, the researchers explained the purpose and significance of the questionnaire and confidentiality principle to the participants.

### Statistical analysis

The qualified research investigators collected the data and numbered the questionnaires uniformly, and two people entered the data using Excel 2019. The study was statistically analyzed using SPSS 26.0 and AMOS 26.0 with confirmatory factor analysis to verify the appropriateness and stability of the scale's construct validity.

#### Item analysis

The central tendency of a question's answer was examined. If an item had one choice in the answer set and the percentage exceeded 80%, then the item was weakly discriminatory and subsequently removed [27, 28]. The Pearson correlation coefficient method was used to remove items that had a low correlation with the total score of the questionnaire (r < 0.30) [29]. The total scores of the questionnaire were arranged in a logical order. In terms of discrimination, the highest 27% of scores were classified as high based on the total score of the PBIQ-C. The lowest 27% were classified as low scores and a two-tailed independent samples t-test was used to analyze the scores of the two groups [30]. Discrimination was good if the scores of both groups reached the significance level (*p* < 0.05) [31].

#### Reliability and validity testing of the questionnaire

This study invited the eight experts to evaluate the relevance of the questionnaire. They used a 4-point Likert scoring system assess the correlation between each item and the questionnaire topic (1 = no correlation to 4 = extremely strong correlation.). We then calculated the content validity index (CVI) of all items and that of the total questionnaire score. Exploratory factor analysis (EFA) was used for the principal component analysis and maximum variance orthogonal rotation. We used Kaiser’s rule of eigenvalues greater than 1 and Cattell’s scree plot method to determine the number of components [32]. Items with lower factor loadings on their primary factor(< 0.40) or the factor to which it belongs contained only one entry of its own (indicating that the entry was independent of other entries) were excluded. The correlation matrix between each dimension and the total score of the questionnaire was tested, and an internal correlation analysis was conducted [27]. Confirmatory factor analysis (CFA) was used to verify the scale structure of the five factors and 21 items obtained by the EFA. CFA was performed to confirm the use of the five-factor PBIQ-C obtained by the EFA. χ2/DF was required to be < 3. The normed fit index (NFI), incremental fit index (IFI), Tucker-Lewis index (TLI), and comparative fit index (CFI) were required to be > 0.9. Additionally, the root mean square error of approximation (RMSEA) was < 0.08, demonstrating good adaptability and model fit [33]. Cronbach’s alpha coefficient and test–retest reliability evaluated the reliability of the questionnaire.

## Results

### Cross-cultural adaptation

Based on the experts’ comments and respondents’ feedback related to content comprehension, language expression habits, cultural background of the questionnaire, and the results of the prediction test, the research group modified the contents as follows: (1) Because the Chinese expression habit was praised and subsequently criticized, the questionnaire began to show a negative evaluation of body image, which would make pregnant women resistant to emotions. Therefore, the reverse questions on items 27–30 on the original scale were placed at the beginning of the questionnaire. Items 27–30 were pre-test versions of items 1–4. (2) The experts suggested adding items about pregnant women’s perceptions of others’ thoughts. Therefor we added item 13, “My husband is satisfied with my figure during pregnancy.” (3) We combined items 10 and 13 from the original questionnaire, keeping item 13 as pre-test version item 16: “During pregnancy, I feel uncomfortable because of my pattern of walking.” Otherwise, this study also combined items 6 and 8 from the original questionnaire as pre-test version item 10: “I feel that I’m not sexually attractive due to lower body obesity during pregnancy.” (4) In the original scale, there was ambiguity in the literal translation of the pre-test version of item 5, “lumbar curve,” and item 10 “the growth of my hips.” Using the conventions of expression in Chinese, these were translated as “waist circumference” and “lower body obesity,” respectively. In the pre-test version of item 9, “the size of my hips” was translated to “the enlargement of my hips. (5) Some items contain direct translations of the original questionnaire. However, Chinese expression habits are euphemistic. Therefore, on the pre-test version of item 5, “ugly” was changed to “unslender.” On the pre-test version item 12, “ridiculous” was changed to “unbeautiful.” On the pre-test version 16 and 18, “shame” was changed to “uncomfortable.” On the pre-test version item 21, “My body is ugly” was changed to “My body is out of shape.” On the pre-test version of item 25, “Due to changes in the genital area during pregnancy, I’m ashamed of my wife during sexual relationship.”, was changed to “I would refuse sex because of changes in the perineum after pregnancy.” On the pre-test version of item 29, “ugly” was changed to “poor.”

### Participants characteristics

A total of 435 questionnaires were distributed to the pregnant women, and 429 questionnaires were returned (effective recovery rate: 98.62%). The 429 participants were randomly divided into two groups using the random number method; 145 participants were used for exploratory factor analysis and 284 participants were used for validation factor analysis. A bachelor’s degree was predominant among the pregnant women, and they primarily lived in urban areas. Most of the women were employees of enterprises. Table 1 summarizes the participants’ demographic characteristics.

**Table 1:** Participants’ demographic characteristics (n = 429)

**Characteristic**		**n(%)**
**Age (years old)**	20–25	61(14.22)
	26–31	204(47.55)
	32–37	142(33.10)
	38–42	22(5.13)
**BMI**	< 18.5	27(6.29)
	18.5 ~ 23.9	218(50.82)
	24.0 ~ 27.9	125(29.14)
	> 28	59(13.75)
**Educational background**	Elementary school	4(0.93)
	Junior high school degree	66(15.38)
	High school degree	33(7.69)
	Junior college	103(24.01)
	Undergraduate degree	182(42.43)
	Master’s degree or above	41(9.56)
**Place of residence**	Urban	233(54.31)
	Rural	196(45.69)
**Profession**	Unemployed	93(21.68)
	Farmers	1(0.23)
	Liberal professions	31(7.23)
	In service staff	15(3.50)
	Employees of enterprises	147(34.27)
	Employees of institutions	88(20.51)
	Else	54(12.58)
**Household Income (CNY**^**a**^**/Month)**	< 2000	1(0.23)
	2000 ~ 4000	36(8.39)
	4000 ~ 6000	116(27.04)
	> 6000	276(64.34)
**Type of pregnancy**	Planned pregnancy	240(55.94)
	Unplanned pregnancy	149(34.73)
	Assisted reproductive reproduction	40(9.33)
**Gestational age**	First trimester(< 12 weeks)	54(12.58)
	Second trimester (13 ~ 28 weeks)	147(34.27)
	Third trimester (> 28 weeks)	228(53.15)

^a^Chinese Yuan

### Item analysis

All items were retained to examine the central tendency of the answers to an item. Critical ration was based on the total score of the PBIQ-C, which ranged from high to low. The top 27% of the scores were grouped into the high-score group, and the bottom 27% of the scores were grouped into the low-score group. In this study, the critical values for the high-score and low-score groups were 90 and 73 respectively, and the scores for each item in the two groups were analyzed using a two-tailed independent sample t-test. Table 2 shows the results of the stand-alone samples t-test comparing the differences in each item between the high- and low-score groups. Items with no statistically significant differences between the two groups were deleted [31]. The results showed that most entries differed significantly between the high-score and low-score groups, with the exception of items 1 and 13.

**Table 2:** Discrimination analysis of the PBIQ-C

The total score was divided into high-score and low-score groups		N	Mean value	Standard deviation	The standard error of the mean	t	Sig
1	1	122	2.00	.81	.07	-.31	0.76
	2	119	2.04	.90	.08	-.31	0.76
2	1	122	1.79	.71	.06	-4.62	0.00
	2	119	2.30	1.00	.09	-4.60	0.00
3	1	122	2.24	.98	.08	-2.56	0.01
	2	119	2.58	1.09	.10	-2.56	0.01
4	1	122	1.87	.74	.07	-3.00	0.00
	2	119	2.18	.85	.08	-3.00	0.00
5	1	122	2.88	1.09	.10	-9.87	0.00
	2	119	4.12	.85	.08	-9.90	0.00
6	1	122	2.10	.83	.07	-19.68	0.00
	2	119	4.14	.78	.07	-19.69	0.00
7	1	122	2.03	.78	.07	-19.15	0.00
	2	119	4.00	.81	.07	-19.14	0.00
8	1	122	2.82	1.19	.11	-11.80	0.00
	2	119	4.28	.64	.06	-11.89	0.00
9	1	122	2.73	1.10	.10	-11.37	0.00
	2	119	4.13	.78	.07	-11.42	0.00
10	1	122	1.93	.72	.07	-17.53	0.00
	2	119	3.79	.91	.08	-17.49	0.00
11	1	122	1.95	.65	.06	-16.57	0.00
	2	119	3.65	.92	.08	-16.50	0.00
12	1	122	2.06	.75	.07	-21.27	0.00
	2	119	4.04	.69	.06	-21.29	0.00
13	1	122	2.39	.90	.08	-1.68	0.09
	2	119	2.59	.97	.09	-1.68	0.09
14	1	122	1.93	.67	.06	-15.97	0.00
	2	119	3.62	.96	.09	-15.90	0.00
15	1	122	1.43	.50	.05	-11.23	0.00
	2	119	2.87	1.31	.12	-11.13	0.00
16	1	122	1.90	.74	.07	-14.31	0.00
	2	119	3.56	1.04	.10	-14.25	0.00
17	1	122	2.58	1.09	.10	-8.81	0.00
	2	119	3.71	.89	.08	-8.83	0.00
18	1	122	2.11	.87	.08	-10.79	0.00
	2	119	3.46	1.07	.10	-10.76	0.00
19	1	122	1.80	.54	.05	-16.07	0.00
	2	119	3.45	1.00	.09	-15.96	0.00
20	1	122	1.79	.63	.06	-16.39	0.00
	2	119	3.55	1.00	.09	-16.30	0.00
21	1	122	1.77	.59	.05	-20.59	0.00
	2	119	3.69	.84	.08	-20.50	0.00
22	1	122	2.15	.92	.08	-14.81	0.00
	2	119	3.86	.88	.08	-14.82	0.00
23	1	122	3.16	.93	.08	2.16	0.03
	2	119	2.89	.98	.09	2.15	0.03
24	1	122	2.09	.73	.07	-13.34	0.00
	2	119	3.50	.91	.08	-13.30	0.00
25	1	122	2.26	.83	.08	-9.68	0.00
	2	119	3.40	.99	.09	-9.65	0.00
26	1	122	2.33	.93	.08	-14.71	0.00
	2	119	3.97	.79	.07	-14.74	0.00
27	1	122	2.40	1.00	.09	-11.59	0.00
	2	119	3.82	.90	.08	-11.61	0.00
28	1	122	2.46	.98	.09	-12.88	0.00
	2	119	3.96	.82	.07	-12.91	0.00
29	1	122	3.00	1.08	.10	-6.64	0.00
	2	119	3.88	.98	.09	-6.65	0.00

Pearson's correlation coefficient was used to assess the correlation between the 29 items and the total questionnaire score. As shown in Table 3, most items were significant with the exception of items 1 and 13. An R value of less than 0.3 indicates a low correlation with the item score and total score; items with an R of less than 0.3 were deleted. Items 1, 2, 3, 4, 13, and 23 had scores less than 0.3 and were thus deleted from the questionnaire.

**Table 3:** Item-total correlation and content validity of the questionnaire

Item	Item Item-to-total correlation	I-CVI	S-CVI
1	-0.02	1	0.81
2	0.18*	1	
3	0.10*	0.88	
4	0.12*	0.88	
5	0.51*	1	
6	0.78*	0.88	
7	0.76*	0.63	
8	0.60*	0.75	
9	0.59*	0.88	
10	0.78*	0.63	
11	0.73*	0.75	
12	0.79*	0.88	
13	0.03	0.75	
14	0.74*	0.63	
15	0.65*	0.63	
16	0.73*	0.75	
17	0.51*	0.63	
18	0.59*	0.75	
19	0.77*	0.75	
20	0.74*	0.75	
21	0.81*	1	
22	0.66*	0.88	
23	-0.15*	0.88	
24	0.72*	0.63	
25	0.61*	0.88	
26	0.71*	0.88	
27	0.61*	0.88	
28	0.66*	0.88	
29	0.41*	0.88	

^*^P < 0.05

### Structural validity

#### Content validity

The content validity index of the items (I-CVI) ranged from 0.63 ~ 1.00, and the content validity index of the scale (S-CVI) was 0.81 (Table 3).

#### Exploratory factor analysis

**Fig. 2:**
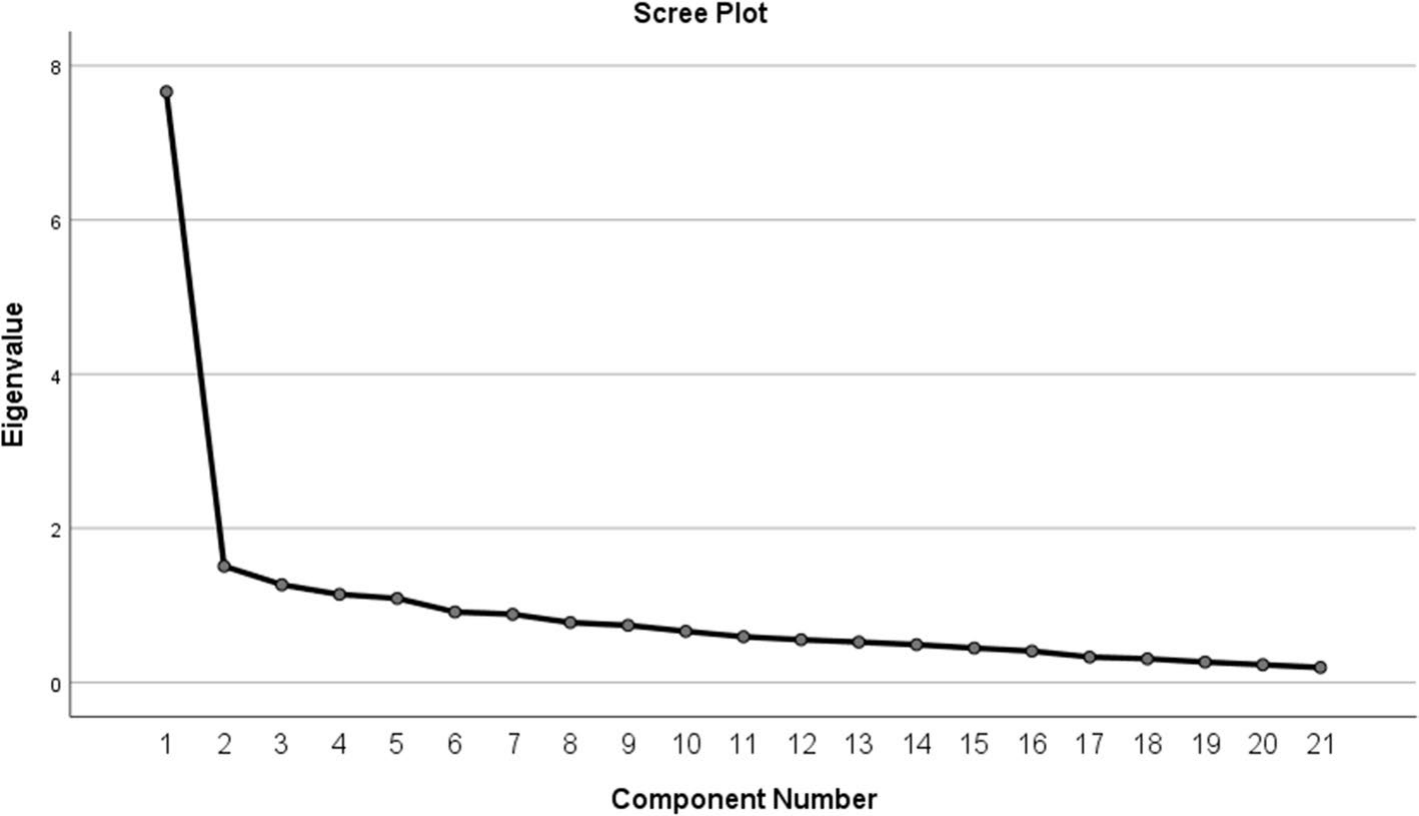
Scree plot for factor components of PBIQ-C

The validity of the questionnaire was assessed in the EFA using Kaiser–Meyer–Olkin (KMO) values, common degree (common factor variance), the variance interpretation rate, load factor, and other indicators. A KMO above 0.8 is reasonable [34]. The KMO value of this study was 0.89 and Bartlett's test of sphericity was significant (chi-square = 1246.42, *p* < 0.01), making it suitable for factor analysis, which indicates the acceptable validity of the questionnaire. The variance explained by the five factors were 16.10% for Factor 1, 13.01% for Factor 2, 12.63% for Factor 3, 11.27% for Factor 4, and 7.33% for Factor 5. And the cumulative variance explained by the rotation was 60.34%, revealing that all information could be extracted effectively. Using Kaiser’s rule of eigenvalues greater than 1 and Cattell’s scree plot method, five factors (eigenvalue > 1) were extracted. The number of factors was also confirmed by the scree plot (Fig. 2). After rotating the component matrix for each question in the questionnaire, each item had a loading coefficient > 0.40 on each dimension, except for item 22 [32]. Only item 17 did constitute a stand-alone factor. Therefore, items 17 and 22 were removed from the questionnaire. The results of EFA are shown in Table 4.

**Table 4:** Factor loading each item in the PBIQ-C (EFA)

Item	Factor (loadings)					Common factor variance
	**1**	**2**	**3**	**4**	**5**	
5	**0.63**	-0.05	0.19	0.18	0.14	0.49
6	**0.84**	0.25	0.11	0.15	-0.01	0.80
7	**0.75**	0.28	0.12	0.08	0.02	0.67
8	**0.49**	0.19	0.27	0.15	0.16	0.40
12	**0.60**	0.13	0.31	0.12	0.17	0.52
14	0.15	**0.75**	0.25	0.09	-0.02	0.65
18	0.01	**0.69**	0.28	0.00	0.16	0.58
19	0.19	**0.60**	0.05	0.34	0.25	0.58
20	0.26	**0.62**	-0.02	0.34	0.02	0.56
21	0.44	**0.61**	0.19	0.29	0.05	0.68
26	0.27	0.32	**0.75**	0.16	0.03	0.76
27	0.21	0.10	**0.80**	0.12	0.05	0.71
28	0.24	0.21	**0.77**	0.16	0.13	0.74
10	0.42	0.15	0.31	**0.49**	0.27	0.60
15	0.12	0.20	0.00	**0.69**	-0.07	0.54
16	0.42	0.31	0.03	**0.48**	0.19	0.53
24	0.27	0.21	0.19	**0.64**	-0.05	0.56
25	0.02	0.02	0.34	**0.67**	0.21	0.61
9	0.39	0.06	0.39	0.15	**0.40**	0.49
11	0.18	0.16	0.25	0.22	**0.67**	0.61
29	0.07	0.06	-0.03	-0.07	**0.76**	0.59
Characteristic root value (before rotation)	7.66	1.51	1.27	1.14	1.09	-
Variance interpretation rate % (before rotation)	36.48	7.18	6.04	5.44	5.19	-
Accumulated variance interpretation rate% (before rotation)	36.48	43.67	49.70	55.15	60.34	-
The characteristic root (after rotation)	3.38	2.73	2.65	2.37	1.54	-
Variance interpretation rate % (after rotation)	16.10	13.01	12.63	11.27	7.33	-
Accumulated variance interpretation rate % (after rotation)	16.10	29.11	41.75	53.01	60.34	-
KMO value	0.89	-	-	-	-	-
Barthes spherical value	1246.42					-
df	210					-
*P*-value	< 0.01					-

#### Confirmatory factor analysis

**Fig. 3:**
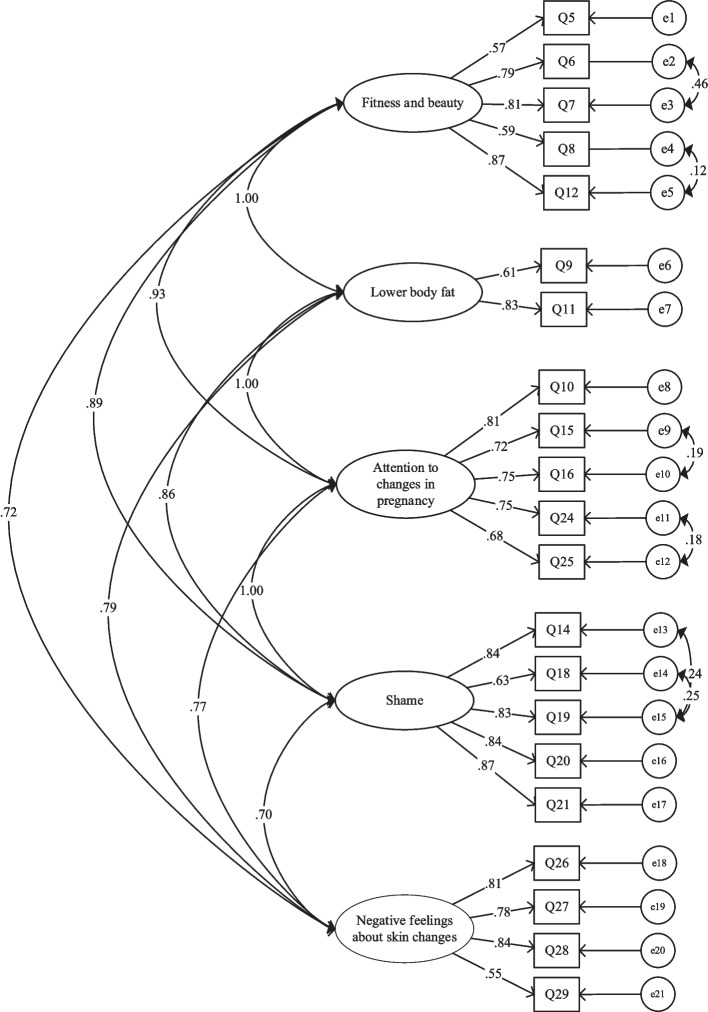
The PBIQ-C model derived from CFA.χ2 = 476.02; df = 173; *p* < 0.01; χ2/df = 2.75

CFA was performed to confirm the five-factor PBIQ-C obtained using EFA [32]. The initial model demonstrated a poor fit to the data (χ2/df = 3.27,* p* < 0.01; NFI = 0.87, IFI = 0.93, TLI = 0.91, CFI = 0.91, and RMSEA = 0.09, *p* < 0.01). Following a review of the suggested modification indices and theoretical framework of the original scale [35], covariance was added between error terms within domains, and item 29 was adjusted from the factor of “lower body fat” to “negative feelings about skin changes.” The modified five-factor model was a good fit for the data (χ2/df = 2.75, *p* < 0.01; NFI = 0.90, IFI = 0.93, TLI = 0.92, CFI = 0.93; RMSEA = 0.079,* p* < 0.01). The standardized factor load of each item was > 0.4. The standardized five-factor structural model of the PBIQ-C (n = 284) is shown in Fig. 3**.**

Our final five-factor solution differs slightly from the seven-factor solution proposed for the original PBIQ. On the PBIQ-C, the “symbol of motherhood” (items 1–4) and “sexual attractiveness” (items 22 and 23) were removed. Based on the results of the exploratory and confirmatory factor analyses, the PBIQ-C merged item 12 into “fitness and beauty” (F1). “Sex attractiveness” was deleted. Items 10, 24, and 25 were merged into “attention to changes in pregnancy” (F3). Item 14 was merged into “shame”(F4). The five final factors included “fitness and beauty” (F1, items 5–8 and 12), “lower body fat”(F2, items 9 and 11), “attention to changes in pregnancy” (F3, items10, 15, 16, 24, and 25), “shame” (F4, items 14 and 18–21) and “negative feelings about skin changes” (F5, items 26–29).

#### Correlation analysis

Correlation analysis was used to examine the correlation between the overall questionnaire and the five dimensions. Pearson’s correlation coefficient was used in order to determine whether there was a good agreement between the total score and subdomain scores on the PBIQ-C. The results of the correlation analysis revealed that the overall questionnaire and the “fitness and beauty,” “lower body fat,” “attention to changes during pregnancy,” “shame,” and “negative feelings toward skin changes” dimensions were all significant. The correlation coefficients for each dimension were 0.87, 0.76, 0.89, 0.88, and 0.75, respectively, indicating the existence of positive correlations between the overall questionnaire and the five dimensions (Table 5).

**Table 5:** Correlation analysis of the five dimensions using Pearson’s correlation coefficient

	Fitness and beauty	Lower body fat	Attention to changes in pregnancy	Shame	Negative feelings about skin changes	The overall questionnaire
Fitness and beauty	1					
Lower body fat	0.70**	1				
Attention to changes in pregnancy	0.73**	0.70**	1			
Shame	0.72**	0.63**	0.83**	1		
Negative feelings about skin changes	0.61**	0.62**	0.63**	0.60**	1	
The overall questionnaire	0.87^**^	0.76^**^	0.89^**^	0.88^**^	0.75^**^	1

^**^ P < 0.01

### Reliability testing

The reliability analysis results showed that the PBIQ-C had ideal internal consistency, with an overall Cronbach’s alpha coefficients of 0.95. The test–retest reliability was 0.80 with a sample size of 25(questionnaire return rate: 100%). The reliability of each dimension of the questionnaire is presented in Table 6.

**Table 6:** Cronbach’s alpha coefficient for each dimension of the questionnaire and deleted items

Dimensionality	Cronbach’s alpha coefficient	Item	Cronbach’s alpha if the item deleted
Fitness and beauty	0.81	5	0.95
		6	0.94
		7	0.94
		8	0.95
		12	0.94
Lower body fat	0.65	9	0.95
		11	0.94
Attention to changes in pregnancy	0.85	10	0.94
		15	0.95
		16	0.94
		24	0.94
		25	0.95
Shame	0.89	14	0.94
		18	0.95
		19	0.94
		20	0.94
		21	0.94
Negative feelings about skin changes	0.81	26	0.94
		27	0.95
		28	0.95
		29	0.95

## Discussion

The maternal body undergoes dramatic changes during pregnancy. These rapid changes may cause a pregnant mother to reevaluate her body image. She may view these changes as natural and caused by pregnancy, and remain satisfied with her body image because of its transitory nature [36]. However, these rapid changes may lead to dissatisfaction with body image. BID is associated with depressive symptoms, unfavorable dieting behaviors, eating disorders, excessive weight gain during pregnancy, and postpartum weight maintenance. Additionally, it is thought to influence parent–child interactions, including problematic child feeding practices, which subsequently may be associated with child self-regulation, eating behaviors, and the risk of childhood obesity [11, 37, 38]. A reliable and valid PBIQ-C will help to more accurately assess and provide insights into the level of body dissatisfaction experienced by Chinese women during different periods of pregnancy. This study adapted and validated the PBIQ-C based on a modified Brislin translation model. The reliability and accuracy of the questionnaire were reviewed based on experts’ opinions. The results showed that the PBIQ-C (comprising the five dimensions of fitness and beauty, lower body fat, attention to changes in pregnancy, shame, and negative feelings about skin changes) provided adequate validity (cross-cultural, structural, and construct validity) and satisfactory internal consistency reliability.

Because of the novelty of the PBIQ and the fact that the factor structure of the Iranian version has only been established in one EFA study thus far [11, 37, 38], we chose an exploratory and confirmatory approach to test the factor structure of the newly developed Chinese version. Compared with the seven-factor structure of the original questionnaire, the EFA of the PBIQ-C revealed a highly interpretable five factor that excluded the “symbols of motherhood” and “sexual attractiveness” dimensions. This five-factor structure was confirmed by CFA and resulted in a more parsimonious scale of 21 items. We chose to omit six items (items 1, 2, 3, 4, 13, and 23) from the final PBIQ-C due to poor a low correlation with the score and lower total score and factor loading on the main factor (item 22).

In the original PBIQ, items 1, 2, 3 and 4 contributed to the factor “symbols of motherhood.” Assisted reproductive technologies (ART) are used to aid in achieving conception in individuals who are diagnosed with infertility [39]. Couples who are diagnosed with infertility have typically tried a variety of methods to become pregnant before they are diagnosed, including good pregnancy preparation and various physical examination. It is only after a diagnosis of infertility is made that they ultimately choose to use ART. A study found that pregnant women who used ART were prepared for pregnancy before pregnancy [40]. In addition, a planned pregnancy is when a woman consciously improves her health before the pregnancy to prepare for pregnancy [41]. Preparation for pregnancy includes psychological and physical preparation, such as the mental attitude to motherhood, selection of the optimum time for becoming pregnant, preventing neural tube defects, and restricting the use of drugs, smoking, etc. [42]. One study has found that preparation for pregnancy improves pregnancy acceptance, and that pregnant women are ready for motherhood [43]. In total, 65.27% of the pregnant women included in this study were those who had prepared for the pregnancy (through a planned pregnancy or ART). Therefore, the assessment of “symbols of motherhood” at this point was of little significance, as pregnant women gradually identify with motherhood after acceptance of the pregnancy. Thus, it is reasonable for us to remove this dimension in the PBIQ-C.

Items 22, 23, 24 and 25 comprised the “sexual attractiveness” factor in the original PBIQ. In our study, items 24 and 25, which asked pregnant women about changes in their breast color and perineum during pregnancy, were incorporated into the third factor, “attention to changes in pregnancy,” and the factor “sexual attractiveness” was removed from the PBIQ-C. It makes sense that the decrease in sexual attraction is ultimately related to physical changes during pregnancy.

This study translated the PBIQ into Chinese for the first time and assessed its reliability and construct validity. We found that the Chinese version of the PBIQ was reliable and suitable for assessing the body image of pregnant Chinese women. Both the PBIQ and the PBIQ-C have shown favorable psychometric properties in terms of reliability. The PBIQ and the PBIQ-C Cronbach’s alpha were 0.925 and 0.95, respectively. In the present study, the Cronbach’s alpha for each dimension of the PBIQ-C was acceptable.

Translation and cross-cultural adaptation processes are essential to ensure that scales are interpreted in the same way across different languages, thus ensuring applicability in several countries where the language is different [44]. Our study translated the PBIQ into the PBIQ-C and adapted it to the Chinese context, ensuring that the items conformed to Chinese cultural expressions. In the pre-test process, pregnant women noted that negative items that arose at the beginning of the questionnaire made them feel uncomfortable. Chinese culture requires a process of adaptation in the face of negative evaluations. Therefore, the research group moved the negative items to the end of the questionnaire. Interestingly, the pregnant women pointed out that they perceived the words to be sharp during the pre-test, which may cause discomfort to feminist pregnant women. Studies have shown that feminism protects women from distorted body image [45, 46]. Feminists are more optimistic about changes in body image. Using harsh language to evaluate bodily changes during pregnancy can cause resentment in pregnant women. Therefore, we used relatively euphemistic expressions for each item without changing its original meaning.

## Limitations

This study has several limitations. First, the present sample was only from one province in China, which may have impacted its generalizability to all Chinese individuals. Therefore, future studies should recruit participants from other provinces in China. Second, some psychometric characteristics of the PBIQ-C, such as its convergent validity, should be assessed further. Third, most of the participants included in this study were in their late pregnancy. Future studies should adopt stratified sampling to enhance representation.

## Conclusion

The PBIQ-C contains 21 items and five dimensions and has a similar theoretical structure to the original questionnaire, with a more satisfactory reliability and validity. All of the PBIQ-C indicators met the measurement requirements, and effectively and scientifically evaluated the body image of pregnant women.

### Supplementary Information

**Additional file 1.** Supplementary materials include the original scale, the translated Chinese version, the use statement of the PBIQ scale. 1. Prenatal body image questionnaire (PBIQ) for pregnant women. 2. Translation scale. The Chinese version of the Prenatal Body Image Questionnaire (PBIQ) for pregnant women.

## Data Availability

The datasets used and analyzed in the current study are available from the corresponding author upon reasonable request.
