# Supplementary material for: Validation and psychometric testing of the Chinese version of the prenatal body image questionnaire
Source: BMC Pregnancy Childbirth. 2024 Feb 1;24:102. doi: 10.1186/s12884-024-06281-w (PMC10835852; doi:10.1186/s12884-024-06281-w)
Supplement: Supplementary file 1 — Additional file 1. Supplementary materials include the original scale, the translated Chinese version, the use statement of the PBIQ scale. 1. Prenatal body image questionnaire (PBIQ) for pregnant women. 2. Translation scale. The Chinese version of the Prenatal Body Image Questionnaire (PBIQ) for pregnant women. [file 12884_2024_6281_MOESM1_ESM.docx]

**Supplementary material**

Supplementary materials include the original scale, the translated Chinese version, the use statement of the PBIQ scale.

**1.Prenatal body image questionnaire (PBIQ) for pregnant women**

| statements | 1 | 2 | 3 | 4 | 5 |
| --- | --- | --- | --- | --- | --- |
| 1. My lumbar curve has an ugly appearance in pregnancy. |  |  |  |  |  |
| 2.Due to changes in pregnancy, I feel that my physical attractiveness is low. |  |  |  |  |  |
| 3.I feel that during pregnancy, my body does not have the feminine elegance. |  |  |  |  |  |
| 4.I’m worried about my fitness, in the postpartum period. |  |  |  |  |  |
| 5.During pregnancy, I try to hide the size of my hips by wearing loose clothes. |  |  |  |  |  |
| 6.I feel that I’m not sexually attractive due to my lower body fat during pregnancy. |  |  |  |  |  |
| 7.Growing my hips during pregnancy has resulted in the loss of my fitness. |  |  |  |  |  |
| 8.I feel that I’m not sexually attractive due to the growth of my hips during pregnancy. |  |  |  |  |  |
| 9.I feel that my lower body fats make my body ridiculous. |  |  |  |  |  |
| 10.I feel that my walking is funny because of my body shape. |  |  |  |  |  |
| 11.During pregnancy, the comments made by others about my appearance have become more important to me. |  |  |  |  |  |
| 12.I will try to hide my facial changes in pregnancy with makeup, even if it is harmful to the fetus. |  |  |  |  |  |
| 13. I’m ashamed of my pattern of walking during pregnancy. |  |  |  |  |  |
| 14. Changes in appearance during pregnancy have attracted the attention of others. |  |  |  |  |  |
| 15. I feel ashamed if strangers see my stomach. |  |  |  |  |  |
| 16. Because of my body shape changes during pregnancy, I prefer not to look at my body in the mirror. |  |  |  |  |  |
| 17. I’m upset that I do not have a beautiful body to wear sexy clothes. |  |  |  |  |  |
| 18. The size of my stomach in pregnancy makes me feel my body is ugly. |  |  |  |  |  |
| 19. I feel my breasts are hanging and deformed. |  |  |  |  |  |
| 20. Growing up my breasts during pregnancy has made me more attractive. (Reverse question) |  |  |  |  |  |
| 21. I feel that my breasts color change during pregnancy has diminished my sexual attractiveness. |  |  |  |  |  |
| 22. Due to changes in the genital area during pregnancy, I’m ashamed of my wife during sexual relationship. |  |  |  |  |  |
| 23. Steria created on my belly skin during pregnancy make me upset. |  |  |  |  |  |
| 24. I’m trying to get rid of my skin steria. |  |  |  |  |  |
| 25. I don’t like people see my belly steria, so I wear clothes that covers my entire body. |  |  |  |  |  |
| 26. I feel ugly with skin changes in pregnancy. |  |  |  |  |  |
| 27. My appearance change in pregnancy makes me feel like a mother (Reverse question). |  |  |  |  |  |
| 28. Because of my interest in pregnancy, I’ve accepted my body-shape changes (Reverse question). |  |  |  |  |  |
| 29. I feel proud of the fact that my body shape during pregnancy indicates my fertility (Reverse question). |  |  |  |  |  |
| 30. I feel relieved that changes in pregnancy can be remedied (Reverse question). |  |  |  |  |  |

Supplementary: In this questionnaire, 5-strongly agree, 4-agree, 3-somewhat agree, 2-disagree, and 1-strongly disagree. A higher score in this questionnaire reflects more dissatisfaction with the BI in pregnancy.

**2. Translation scale：**

The Chinese version of the Prenatal Body Image Questionnaire (PBIQ) for pregnant women.

|  | 1  strongly disagree | 2  disagree | 3  somewhat agree | 4  agree | 5  strongly agree |
| --- | --- | --- | --- | --- | --- |
| 1. My waist circumference was unslender during pregnancy. |  |  |  |  |  |
| 2. Due to changes in pregnancy, I feel that my physical attractiveness is low. |  |  |  |  |  |
| 3. I feel that my body does not have feminine elegance during pregnancy. |  |  |  |  |  |
| 4. I’m worried about my fitness, in the postpartum period. |  |  |  |  |  |
| 5. I think the obesity of my lower body makes my body look unbeautiful. |  |  |  |  |  |
| 6. During pregnancy, I try to hide the enlargement of my hips by wearing loose clothes. |  |  |  |  |  |
| 7. Growing my hips during pregnancy has resulted in the loss of my fitness. |  |  |  |  |  |
| 8. I feel that I’m not sexually attractive due to lower body obesity during pregnancy. |  |  |  |  |  |
| 9. I will try to hide my facial changes in pregnancy with makeup, even if it is harmful to the fetus. |  |  |  |  |  |
| 10. During pregnancy, I feel uncomfortable because of my pattern of walking. |  |  |  |  |  |
| 11. I feel that my breast's color change during pregnancy has diminished my sexual attractiveness. |  |  |  |  |  |
| 12. I would refuse sex because of changes in the perineum after pregnancy. |  |  |  |  |  |
| 13. During pregnancy, the comments made by others about my appearance have become more important to me. |  |  |  |  |  |
| 14. I feel uncomfortable if strangers see my stomach. |  |  |  |  |  |
| 15. Because of my body shape changes during pregnancy, I prefer not to look at my body in the mirror. |  |  |  |  |  |
| 16. I’m upset that I do not have a beautiful body to wear sexy clothes. |  |  |  |  |  |
| 17. The size of my stomach in pregnancy makes me feel my body is out of shape. |  |  |  |  |  |
| 18. Steria created on my belly skin during pregnancy makes me upset. |  |  |  |  |  |
| 19. I’m trying to get rid of my skin steria. |  |  |  |  |  |
| 20. I don’t like people to see my belly steria, so I wear clothes that cover my entire body. |  |  |  |  |  |
| 21. I feel poor with skin changes in pregnancy. |  |  |  |  |  |

Supplementary：A higher score in this questionnaire reflects more dissatisfaction with the BI in pregnancy.
